# A jingmenvirus RNA-dependent RNA polymerase structurally resembles the flavivirus counterpart but with different features at the initiation phase

**DOI:** 10.1093/nar/gkae042

**Published:** 2024-01-31

**Authors:** Xinyu Wang, Xuping Jing, Junming Shi, Qiaojie Liu, Shu Shen, Peter Pak-Hang Cheung, Jiqin Wu, Fei Deng, Peng Gong

**Affiliations:** Key Laboratory of Special Pathogens and Biosafety, Wuhan Institute of Virology, Center for Biosafety Mega-Science, Chinese Academy of Sciences, No. 262 Jin Long Street, Wuhan, Hubei 430207, China; University of Chinese Academy of Sciences, Beijing 100049, China; Key Laboratory of Special Pathogens and Biosafety, Wuhan Institute of Virology, Center for Biosafety Mega-Science, Chinese Academy of Sciences, No. 262 Jin Long Street, Wuhan, Hubei 430207, China; University of Chinese Academy of Sciences, Beijing 100049, China; State Key Laboratory of Virology, Wuhan Institute of Virology, Center for Biosafety Mega-Science, Chinese Academy of Sciences, No.262 Jin Long Street, Wuhan, Hubei 430207, China; Key Laboratory of Special Pathogens and Biosafety, Wuhan Institute of Virology, Center for Biosafety Mega-Science, Chinese Academy of Sciences, No. 262 Jin Long Street, Wuhan, Hubei 430207, China; State Key Laboratory of Virology, Wuhan Institute of Virology, Center for Biosafety Mega-Science, Chinese Academy of Sciences, No.262 Jin Long Street, Wuhan, Hubei 430207, China; Department of Chemical Pathology, Chinese University of Hong Kong, Prince of Wales Hospital, Shatin, New Territories, Hong Kong, China; Key Laboratory of Special Pathogens and Biosafety, Wuhan Institute of Virology, Center for Biosafety Mega-Science, Chinese Academy of Sciences, No. 262 Jin Long Street, Wuhan, Hubei 430207, China; State Key Laboratory of Virology, Wuhan Institute of Virology, Center for Biosafety Mega-Science, Chinese Academy of Sciences, No.262 Jin Long Street, Wuhan, Hubei 430207, China; Key Laboratory of Special Pathogens and Biosafety, Wuhan Institute of Virology, Center for Biosafety Mega-Science, Chinese Academy of Sciences, No. 262 Jin Long Street, Wuhan, Hubei 430207, China; Drug Discovery Center for Infectious Diseases, Nankai University, Tianjin 300350, China

## Abstract

Jingmenviruses are a category of emerging segmented viruses that have garnered global attention in recent years, and are close relatives of the flaviviruses in the *Flaviviridae* family. One of their genome segments encodes NSP1 homologous to flavivirus NS5. NSP1 comprises both the methyltransferase (MTase) and RNA-dependent RNA polymerase (RdRP) modules playing essential roles in viral genome replication and capping. Here we solved a 1.8-Å resolution crystal structure of the NSP1 RdRP module from Jingmen tick virus (JMTV), the type species of jingmenviruses. The structure highly resembles flavivirus NS5 RdRP despite a sequence identity less than 30%. NSP1 RdRP enzymatic properties were dissected in a comparative setting with several representative *Flaviviridae* RdRPs included. Our data indicate that JMTV NSP1 produces characteristic 3-mer abortive products similar to the hepatitis C virus RdRP, and exhibits the highest preference of terminal initiation and shorter-primer usage. Unlike flavivirus NS5, JMTV RdRP may require the MTase for optimal transition from initiation to elongation, as an MTase-less NSP1 construct produced more 4–5-mer intermediate products than the full-length protein. Taken together, this work consolidates the evolutionary relationship between the jingmenvirus group and the *Flaviviridae* family, providing a basis to the further understanding of their viral replication/transcription process.

## Graphical Abstract

**Graphical Abstract:**
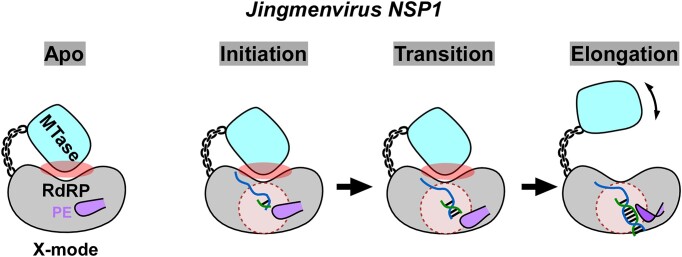

## Introduction

Jingmenviruses represent a cluster of newly identified positive-strand RNA viruses that exhibit the closest evolutionary relationship to viruses belonging to the *Flavivirus* genus and *Flaviviridae* family. Consequently, they are commonly referred to as flavi-like viruses (1–3). The Jingmen tick virus (JMTV) is classified as the type species of jingmenviruses. It was initially discovered in a tick pool of *Rhipicephalus microplus* (3) collected in the Jingmen region of Hubei province, China (1). Between the years 2014 and 2016, a number of jingmenviruses exhibiting similarities were discovered in various regions of China. While it was previously known that these viruses could replicate in mammalian cells and arthropod cell lines (3), there was no evidence of human infection until a jingmenvirus of the Kosovo type was detected in the serum of patients infected with Crimean-Congo hemorrhagic fever virus in 2018 (4). Similarly, Alongshan virus was found in samples from patients who exhibited fever or had been bitten by ticks in northeast China (5). Thus far, numerous jingmenviruses, which are frequently designated based on their geographical origin, have been identified in diverse arthropod specimens from various locations such as Trinidad, Laos, Finland, Guinea, France, and Brazil, encompassing the continents of Eurasia, Africa, and America (6). The present list of confirmed hosts for jingmenvirus encompasses arthropods such as ticks, mosquitoes, fleas, and crickets, as well as mammals including bats, rats, cows, monkeys, and humans. The jingmenviruses exhibit a noteworthy capacity for host range expansion, global distribution, and pose significant risks to human health. Consequently, these emerging viruses warrant considerable attention and are of utmost importance for fundamental scientific investigation.

**Figure 1.**
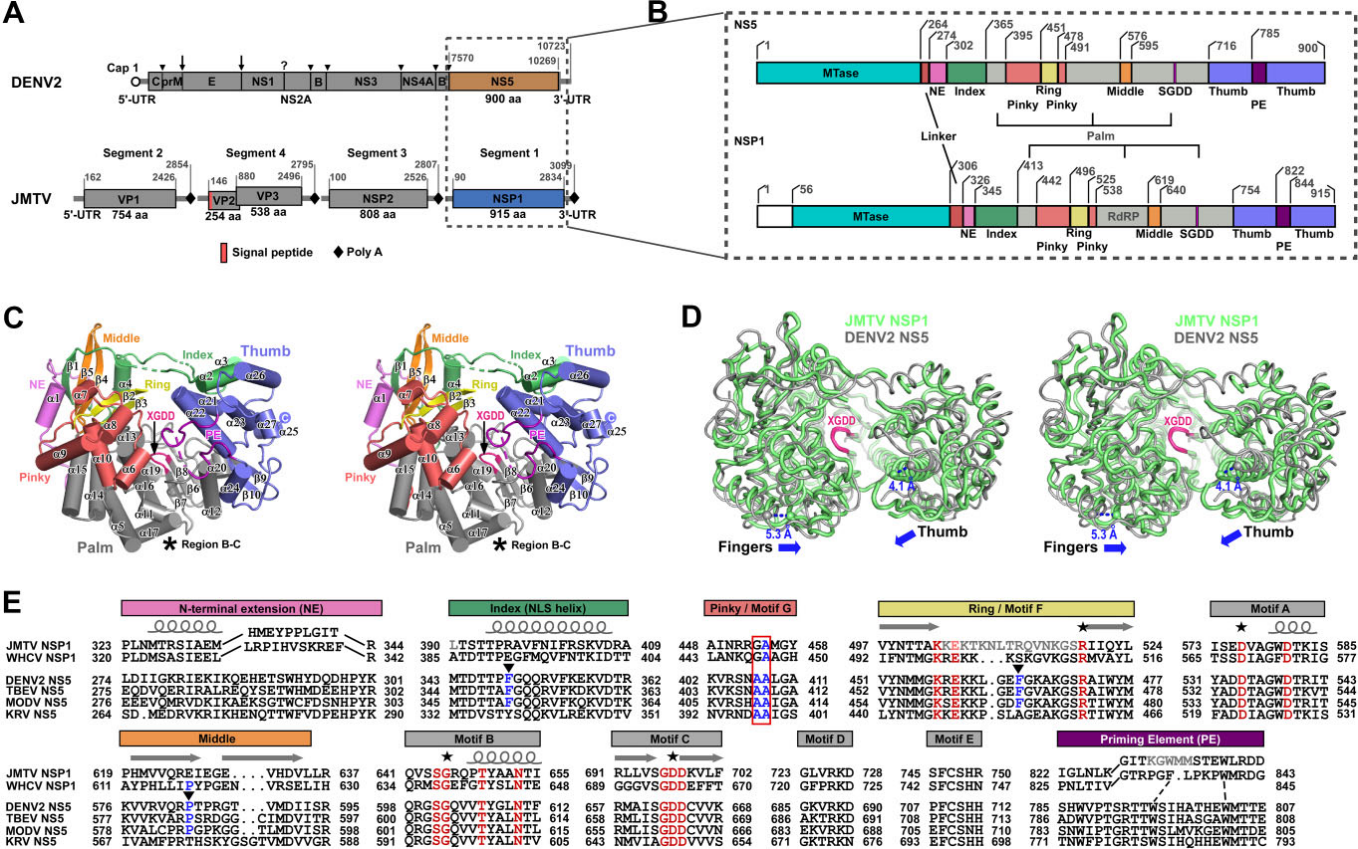
Global views of the JMTV RdRP structure. (**A**) A comparison of DENV2 and JMTV genome organization. UTR: untranslated region; aa: amino acid residue. (**B**) A color-coded bar showing structural elements for JMTV NSP1 and DENV2 NS5. The sequence identity for these two protein is 25% and it is noteworthy that NSP1 contains an additional 55 residues at its N-terminus. Coloring scheme: MTase in cyan, palm in grey, thumb in slate, linker in red, NE in violet, index in green, middle in orange, ring in yellow, pinky in light red, PE in purple, and signature XGDD sequence of motif C in magenta. (**C**) Stereo-pair view of JMTV RdRP crystal structure viewing from the dsRNA exiting channel (front view) following the same color coding as in panel B. (**D**) A superimposition of JMTV RdRP (light green, 8WIM, this work) and DENV2 RdRP (grey, PDB entry: 6IZY) viewing from the top (RMSD for all superimposable α-carbon atoms: 2.2 Å, 84.6% residue coverage, NSP1 structure as the reference). Blue arrows indicate the difference in relative placement of thumb and fingers domains in these two structures. (**E**) Structure-assisted sequence alignment of motifs A-G and other important elements. JMTV and WHCV (Wuhan cricket virus) are chosen to represent tick-borne and arthropod-specific jingmenviruses, respectively; DENV2, TBEV, MODV (Modoc virus), and KRV (Kamiti River virus) represent mosquito-borne, tick-borne, vector-unknown, and arthropod-specific flaviviruses, respectively. Four invariant residues in viral RdRPs are indicated by asterisks. Highly conserved residues in motifs A/B/C/F are colored in red. Blue letters combined with red box indicate two key motif G residues in regulating translocation. Blue letters labeled by solid triangle indicate key hydrophobic residues involved in JEV-mode MTase-RdRP interactions. Dashed lines indicate possible conservation of key aromatic residues in PE. Letters in grey indicate residues that are unresolved in electron density maps of the crystal structure. Springs and block arrows on top of the sequences indicate conserved α-helices and β-strands, respectively. Color bars at top of the alignment match the color coding in panel B excluding the YGDD sequence.

In contrast to flaviviruses such as dengue virus (DENV) and Japanese encephalitis virus (JEV), which possess a nonsegmented genome of approximately 10–11 kilobases and encode seven replication proteins (NS1, NS2A, NS2B, NS3, NS4A, NS4B and NS5) (7), jingmenviruses typically exhibit 4–5 genome segments of similar total size. These jingmenviruses only encode two replication proteins, namely NSP1 and NSP2, which share homology with flavivirus NS5 and NS2B-NS3, respectively (1,8). Notably, there is no observed homology between the structural proteins responsible for forming the virus particles in these two virus groups (Figure 1A, B). Therefore, the investigation of the two jingmenvirus replication proteins has been crucial in elucidating their significant connections to their flavivirus counterparts. The flavivirus replication/transcription complex (RTC) consists of NS5 and NS3 as its central components. NS5 is composed of an N-terminal methyltransferase (MTase) and a C-terminal RNA-dependent RNA polymerase (RdRP), while NS3 contains an N-terminal protease that interacts with NS2B and a C-terminal NTPase/helicase (9–11). Multiple studies have provided evidence for the coordination between NS5 and NS3 in viral replication, transcription, and viral genome capping (12–15). Additionally, the NS2B-NS3 protease has been found to play crucial roles in viral polyprotein processing (16,17). The RdRP enzyme module stands out as the sole conserved component across all RNA viruses (18). Consequently, it represents an optimal target for the development of antiviral drugs. The core structure of viral RdRPs exhibits a resemblance to a cupped human right hand, consisting of palm, fingers, and thumb domains that surround the active site. The fingertips of the RdRPs typically interact with the top of the thumb, forming an encirclement (19,20). The classification of viral RdRP can be based on initiation modes, specifically into two groups: primer-dependent and *de novo*. Prominent instances of the former category encompass poliovirus 3D^pol^ (21) and severe and acute respiratory syndrome coronavirus 2 (SARS-CoV-2) nsp12–nsp7–nsp8 (22,23). Conversely, flavivirus NS5 and bacteriophage phi6 polymerase serve as exemplars of the latter (7,24,25). The priming element (PE), typically found within the RdRP thumb, is of significant importance during initiation. Its role is believed to involve the precise placement of the template and the stabilization of the initiation nucleotides (25,26). It has been recently discovered that the *Flaviviridae* RdRP undergoes a transition from initiation to elongation when a 5–6 nt RNA product is synthesized. Additionally, the PE undergoes a refolding process in order to interact with the lengthened template–product RNA duplex, thereby enhancing the stability of the elongation complex (24). Moreover, despite less than 30% sequence identity to flavivirus NS5, JMTV NSP1 has the same layout of MTase-RdRP and a similar size of about 900 residues. Two global conformation modes have been identified in flavivirus NS5 with the JEV-mode conformation critical for RdRP initiation activities and the DENV3 (DENV serotype 3)-mode conformation relevant in promoter- and NS3-binding (11,15,27). However, whether jingmenvirus NSP1 follows similar mechanisms in RdRP synthesis and how does the MTase of NSP1 regulate RdRP synthesis both remain to be clarified.

In this study, we successfully obtained crystal structures of NSP1 RdRP at a resolution of up to 1.84-Å by crystallizing two truncation mutants of JMTV NSP1. The obtained structures provide a comprehensive depiction of the high-resolution structural characteristics of the jingmenvirus group's RdRP. The structural characteristics of the majority of the mentioned entity bear a strong resemblance to those observed in flavivirus RdRPs. This resemblance is particularly evident in the presence of two distinct elements that are considered hallmarks of flaviviruses. These findings provide further evidence of the evolutionary proximity between the RdRPs of the two virus groups, despite the relatively low level of sequence identity. The data from enzymology studies reveal several distinctions between JMTV NSP1 and DENV2 NS5, a representative flavivirus RdRP. During the synthesis of longer target products, 3-mer abortive products are notably observed. Additionally, the elimination of the MTase module in NSP1 leads to elevated levels of intermediate products. This suggests that the MTase plays a crucial role in facilitating a smooth transition from initiation to elongation, thereby promoting efficient enzymatic activity. Furthermore, it has been observed that JMTV NSP1 exhibits a higher level of stringency in terms of initiation from the terminal of the RNA template. Additionally, it has been found that JMTV NSP1 tends to preferentially utilize a dinucleotide primer over longer primers. The structural resemblance and functional difference on one hand refine the understandings of the evolutionary relationship of the two virus groups, and on the other hand make jingmenvirus-flavivirus an ideal duo-system in studying *de novo* viral RdRPs and their intervention strategies.

## Materials and methods

### Plasmid construction and protein expression

The pET26b-JMTV-NSP1 plasmid was generated by cloning the full-length JMTV NSP1 gene (strain: YN-flaviV, genbank entry: MH814977) into the pET26b vector. The FL NSP1 plasmid served as the template for generating mutant plasmids to produce two N-terminal truncation mutants, namely D55 and D307 (representing residues 56–915 and 308–915, respectively). This was achieved using the QuikChange site-directed mutagenesis method (28). The *Escherichia coli* strain Rosetta(DE3) was used to transform each plasmid for the purpose of expressing NSP1 or its variant, both of which were equipped with a C-terminal GSSSGTHHHHHH tag. The cells were cultivated overnight at a temperature of 37°C and a rotational speed of 180 revolutions per minute (rpm) in LB medium supplemented with 100 μg/ml kanamycin (KAN) and 20 μg/ml chloramphenicol (CHL) until the optical density at a wavelength of 600 nm (OD600) reached a value of 1.0. This starter culture was utilized for inoculating a 1-l LB medium supplemented with 100 μg/ml KAN and 20 μg/ml CHL. The aim was to achieve an initial OD600 of approximately 0.025. The cells were cultivated at a temperature of 37°C and 200 rpm until the OD600 reached 1.0. Subsequently, the cells were cooled to the room temperature. Isopropyl-β-d-thiogalactopyranoside (IPTG) was added to reach a final concentration of 0.5 mM for induction and the cells were cultivated at a temperature of 16°C and a speed of 180 rpm for an additional duration of 20 h prior to collection. The protein expression protocols utilized for the FL DENV2 NS5 construct, its N-terminal truncation mutant D263 (representing residues 264–900), hepatitis C virus (HCV) NS5B, and classical swine fever virus (CSFV) NS5B are consistent with those previously documented (24,29).

### Purification of JMTV NSP1 and its truncation mutants

The procedures for cell lysis, protein purification, and protein storage were carried out following the methods described in a previous study on JEV NS5 (11), with minor modifications. Specifically, Tris (pH 7.5) was utilized in the GF buffer for gel-filtration chromatography, while 500 mM NaCl was employed in the GF buffer for the D307 construct. The molar extinction coefficient for NSP1 constructs was determined by utilizing the ExPASy ProtParam program (https://web.expasy.org/protparam/). The typical yield of pure protein per liter of bacterial culture ranges from 1 to 5 milligrams. The expression and purification of a D55 mutant protein, in which selenomethionine (Se-Met) was substituted, was carried out using established methods. The protein was expressed under conditions that inhibit the methionine-biosynthesis pathway, and purification was performed following the identical procedure used for the native protein. This Se-Met-substituted D55 mutant was utilized for phase estimation in the process of structure determination. The protein purification protocols for FL DENV2 NS5, its D263 mutant, HCV NS5B, and CSFV NS5B are consistent with previously described methodologies (24,29).

### Protein crystallization, diffraction data collection and structure determination

Flake-shaped crystals of JMTV NSP1 D55 were successfully obtained within a span of 15 days through the utilization of the sitting drop vapor diffusion method at a temperature of 16°C. Protein concentrations of 12 and 14 mg/ml were employed during the experiment. In the standard procedure, a protein solution in GF buffer containing 5 mM tris-(2-carboxyethyl) phosphine (TCEP) was combined with an equivalent volume of a precipitant/well solution consisting of 0.2 M magnesium chloride (MgCl_2_), 0.1 M HEPES (pH 7.5), and 25% (v/v) polyethylene glycol (PEG) 3350. The protein solution volume used was typically 0.4 μl. A crystal of D307 with a stick-like shape was successfully obtained within a period of 3 days, employing the identical methodology as that employed for D55. The crystal was grown in a precipitant/well solution containing a concentration of 0.4 M potassium sodium tartrate tetrahydrate. The crystals were subjected to a cryo-protectant solution, which consisted of the corresponding mother liquor supplemented with 20% (v/v) glycerol. This was achieved by gradually exchanging the buffer before rapidly cooling the crystals in liquid nitrogen.

The X-ray diffraction data sets were obtained from the Shanghai Synchrotron Radiation Facility (SSRF) beamlines BL02U1 (D307 construct) and BL19U1 (D55 construct). The data was collected using a wavelength of 0.9792 Å and at a temperature of 100 K. Typically, data collection involved a range of at least 360°, with oscillation steps of 0.2–0.5°. The process of integrating, merging, and scaling reflections was carried out using HKL2000 software, as shown in Table 1. Structures of RdRP region of flavivirus NS5 (e.g. JEV NS5 structure, PDB entry: 4K6M) were used as the search model in molecular replacement trials, but did not yield successful solutions. The structure of Se-Met_D55, which represents approximately 75% of the final model residues, was initially determined using a single-wavelength anomalous diffraction (SAD)-based method. This was achieved by employing AutoSol and AutoBuild modules in the PHENIX Suite, which allowed the identification of Se atom sites, estimation of phase information, density modification, and automated model building (30,31). Several iterations of model building and refinement were conducted using the Coot and PHENIX (30). The final Se-Met_D55 model was used as the search model in molecular replacement trials by Phaser to solve the native structures of D55 and D307 (32). The generation of 3500 K composite simulated-annealing (SA) omit 2*F*_o_ – *F*_c_ electron density maps was carried out using the Crystallography & NMR System (CNS) (33). All structure superimpositions were conducted using the maximum-likelihood superpositioning program THESEUS with all structures superimposed simultaneously (34).

**Table 1.** X-ray diffraction data collection and structure refinement statistics

Crystal PDB entry	D55 (Se-Met)	D55 (native) 8WIL	D307 (native) 8WIM
**Data collection** ^a^			
Space group	*P*2_1_2_1_2_1_	*P*2_1_2_1_2_1_	*P*2_1_2_1_2_1_
Cell dimensions			
*a*, *b*, *c* (Å)	61.7, 88.4, 122.4	61.5, 89.2, 121.8	61.5, 89.3, 122.4
α, β, γ (ˆ)	90, 90, 90	90, 90, 90	90, 90, 90
Resolution (Å)^b^	50.0–2.31 (2.39–2.31)^b^	60.0–2.10 (2.18–2.10)^b^	50.0–1.84 (1.91–1.84)^b^
*R*_merge_	0.124 (0.531)	0.069 (0.447)	0.077 (0.583)
*R*_meas_	0.132 (0.569)	0.098 (0.522)	0.083 (0.671)
CC_1/2_	1.000 (0.890)	1.000 (0.968)	0.999 (0.772)
I/σI	17.4 (3.0)	33 (6.2)	22.5 (2.3)
Completeness (%)	99.1 (95.8)	100 (99.9)	99.1 (93.5)
Redundancy	9.4 (7.7)	18.9 (19.1)	6.7 (3.6)
**Structure refinement**			
Resolution (Å)		2.10	1.84
*R*_work_/*R*_free_^c^ (%)		21.5/25.1	19.0/21.7
No. reflections		40074	58904
No. atoms			
Protein		4515	4534
Ligand/ion/water		57/1/383	38/1/419
*B*-factors (Å^2^)			
Protein		27.7	25.2
Ligand/ion/water		44.4/30.7/34.1	33.8/17.8/32.1
R.m.s. deviations			
Bond lengths (Å)		0.008	0.008
Bond angles (°)		0.851	0.916
Ramachandran statistics^d^		92.4/6.8/0.6/0.2	93.6/6.2/0.0/0.2

^a^One crystal was used for data collection for each structure.

^b^Values in parentheses are for the highest-resolution shell.

^c^5% of data are taken for the *R*_free_ set, and the same *R*_free_ set is applied for the all structures.

^d^Values are in percentage and are for most favored, additionally allowed, generously allowed, and disallowed regions in Ramachandran plots, respectively.

### RNA preparation

The *de novo* RdRP assays utilized chemically synthesized RNA templates, specifically the 30-mer (T30 and T30-7) and 28-mer (T28) RNA molecules. These templates were obtained from Integrated DNA Technologies or Dharmacon and featured a hairpin structure at their 5′-region. The template RNA molecules with lengths of 31 nucleotides (T31), 32 nucleotides (T32), and 34 nucleotides (T34) were synthesized using a method involving T7 RNA polymerase and the glmS ribozyme, as described in previous studies (35,36). The RNA was purified using 12% (w/v) polyacrylamide/7M urea gel electrophoresis. Subsequently, it was excised from the gel, eluted using an EluTrap device (GE Healthcare), ethanol precipitated, and finally stored at a temperature of −80°C. Prior to storage, the RNA underwent a self-annealing process, which was previously described (36). The 5′-phosphorylated dinucleotide pGG (P2) from Jena Biosciences was subjected to annealing with the template RNA at molar ratios of 5:1 or 20:1. This annealing process involved a 3-min incubation at 45°C, followed by gradual cooling to room temperature in an RNA annealing buffer containing 50 mM NaCl, 5 mM MgCl_2_, and 5 mM Tris (pH 7.5). As a result, the T28/P2, T30/P2, T30-7/P2, T31/P2, T32/P2 and T34/P2 RNA constructs were formed. The trinucleotide pGGA (P3) and tetranucleotide pGGAU (P4) (obtained from Takara Bio) were phosphorylated at the 5′ position and subsequently annealed with T30 in a 5:1 molar ratio. This resulted in the formation of T30/P3 and T30/P4 constructs, using the same annealing procedure as the constructs containing P2. Note that, the annealing process of P2/P3/P4 and template RNA may not be necessary due to low melting temperature corresponding to only 2–4 bp.

### *In vitro* RdRP assays

A standard reaction mixture of 20 μl was prepared, containing the following components: 4 μM RNA construct (based on template concentration), 6 μM RdRP, 300 μM ATP, 300 μM UTP, 50 mM Tris (pH 7.5), 20 mM NaCl, 5 mM MgCl_2_ and 5 mM dithiothreitol (DTT). The mixture was then incubated at a temperature of 30°C for varying durations. To halt the reaction, an equal volume of stop solution consisting of 95% (v/v) formamide, 20 mM EDTA (pH 8.0), and either 0.02% (w/v) bromphenol blue or 0.02% (w/v) xylene cyanol was added. It is important to note that in the CSFV NS5B and HCV NS5B reactions, Tris (pH 7.0) and MES (pH 6.5) were employed as the buffering agents, respectively. In JMTV assays, the molar ratio of P2 to template is 20:1, which is utilized to investigate the transition from initiation to elongation. Similarly, this molar ratio is also employed in both JMTV and DENV2 assays to evaluate terminal preference. Alternatively, the ratio between P2 and template is 5:1. The denaturing polyacrylamide gel electrophoresis, gel staining, and quantification procedures employed in this study were consistent with those outlined in a previous investigation on DENV2 NS5 (37).

## Results

### JMTV NSP1 RdRP structurally largely resembles its flavivirus counterpart

To gain insights into the structural characteristics of jingmenvirus NSP1, we conducted a crystallization screening experiment for three variants: FL (residues 1–915) JMTV NSP1, as well as two N-terminal truncation constructs, namely D55 (residues 56–915) and D307 (residues 308–915). Although the FL construct did not yield any crystals, the D55 and D307 constructs produced crystals with flake-like and stick-like shapes, respectively. The initial determination of the structure of D55 involved the utilization of the SAD method, wherein Se-Met-substituted D55 crystals were employed. Subsequently, the structures of native D55 and D307 were resolved through molecular replacement, utilizing the Se-Met-derived D55 structure as the search model (Table 1). All three structures are isomorphous with the same *P*2_1_2_1_2_1_ space group cell dimensions. Each crystallographic asymmetric unit consists of a single NSP1 monomer. The D55 crystal was found to be an N-terminal degraded D55 protein (Supplementary Figure S1), but the precise N-terminus of the protein in the crystal was not determined. However, electron density was not detected for residues 56–302 in both the native and Set-Met-derived D55 structures. Due to the significant similarity between the native structures of D55 and D307, with a root-mean-square deviation (RMSD) value of merely 0.2 Å for all superimposable α-carbon atoms (with D307 structure as the reference, covering 99.5% of residues), we primarily utilize the 1.84-Å D307 structure for subsequent illustrations.

The NSP1 RdRP exhibits a characteristic architectural feature commonly found in viral RdRPs, known as encircled right-hand architecture. This architecture consists of distinct domains, namely fingers, palm, and thumb. The NSP1 RdRP closely resembles the RdRPs found in the *Flavivirus* genus, which includes the N-terminal extension (NE) and one-segment PE, both of which are characteristic features of flaviviruses (Figure 1C–E). The active site is situated within the interstitial region of the three domains, encompassed by seven RdRP catalytic motifs A-G (38). Even if compared with the most resembling flavivirus RdRP structure obtained using an MTase-less DENV2 NS5 (39), the right-hand structure of JMTV NSP1 RdRP exhibits a tighter grip with the top of both thumb and fingers domains (primarily pinky finger) moving inward for 4–5 Å (Figure 1D). This phenomenon has been previously observed in picornavirus RdRPs during the formation of an elongation complex (40). The crystal packing interactions may only contribute to the restriction of thumb but not the pinky finger (Supplementary Figure S2). Therefore, the tighter grip observed in these NSP1 structures is likely not a crystallization artifact. The individual finger subdomain nomenclature, initially proposed in PV RdRP, is utilized in order to facilitate the elucidation of structural characteristics of RdRP (20). The D307 structure exhibits unresolved regions, namely residues 359–361 and 388–389 in the index finger, residues 504–518 at the tip of the ring finger, residues 779–785 in the thumb loop located between α-helices 21 and 22, residues 830–836 in part of PE, and residues 912–915 at the very C-terminus. The *Flaviviridae* family exhibits a high degree of conservation in residues found in motifs A, B, C and F. These residues, which are also observed in NSP1 (as shown in Figure 1E, highlighted in red), include four RdRP-invariant residues (as indicated by asterisks in Figure 1E) (41). The RdRP translocation process is regulated by two motif G residues, as documented previously (42). In this context, the flavivirus alanine-alanine combination is substituted by glycine-alanine, which is equivalent to the pestivirus sequence. This substitution is visually represented in Figure 1E, where the motif G residues are highlighted by a red box. The FL JEV NS5 structure, as identified in previous studies (11,37,38), reveals the involvement of three key hydrophobic residues in MTase interactions. These residues, indicated by triangles in Figure 1E, do not exhibit obvious conservation. This lack of conservation suggests that the intra-molecular interaction mode between the jingmenvirus MTase and RdRP, if it exists, may differ from the observed modes in flaviviruses, as reported previously (11,15,27,37).

### The JMTV NSP1 RdRP exhibits several noteworthy substructure features

**Figure 2.**
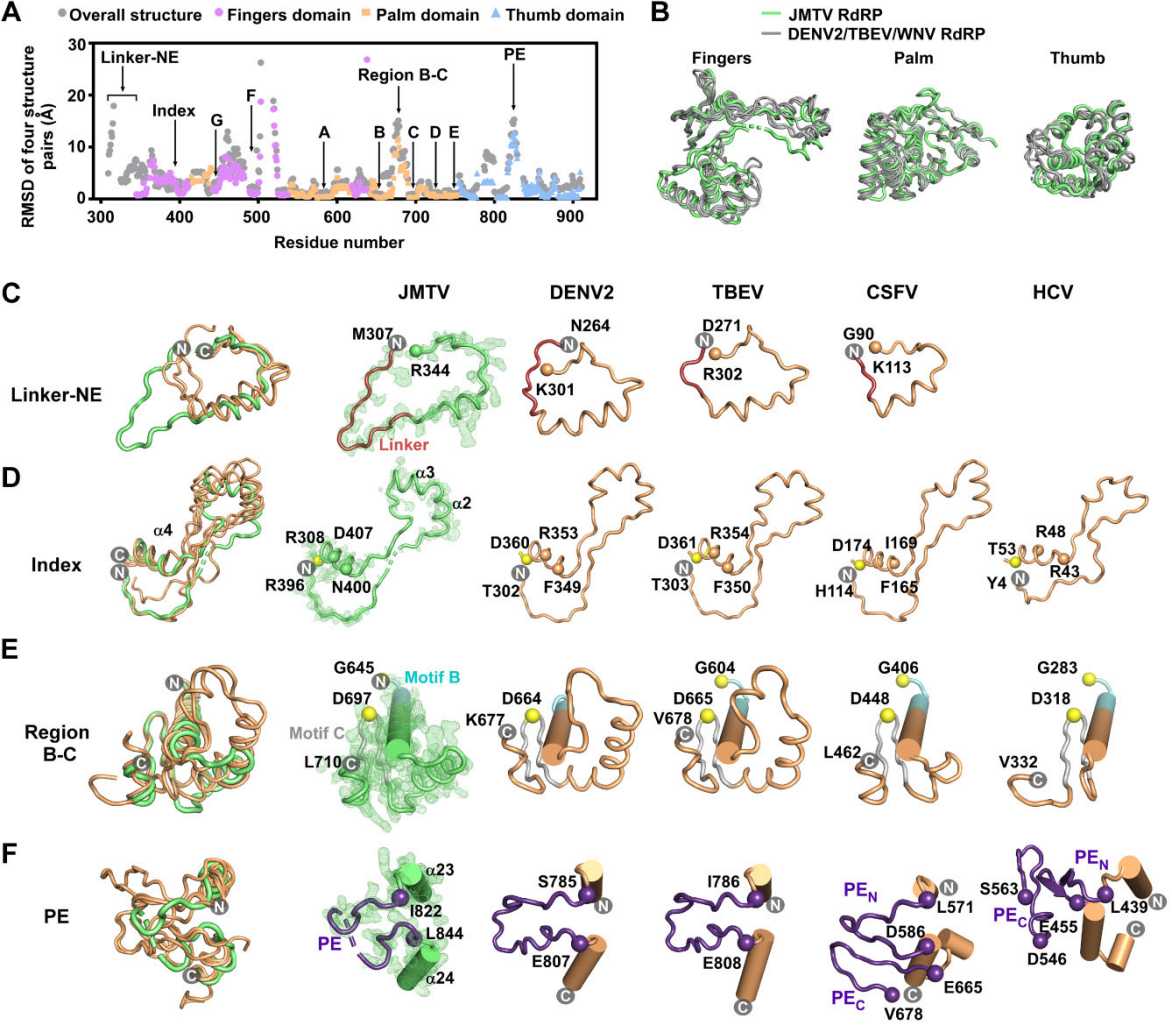
The structural diversity in JMTV and *Flaviviridae* RdRPs. (**A**) A diagram showing the structural similarities of RdRP of JMTV and three representative flaviviruses. Entire RdRP-based, palm-based, fingers-based, and thumb-based RMSD values are shown as grey, orange, pink, and light blue points. (**B**) Superimposition of individual domains for JMTV (green, PDB entry: 8WIM, this work), DENV2 (grey, PDB entry: 6IZY), TBEV (PDB entry: 7D6N) and WNV (PDB entry: 2HFZ) RdRPs. (C–F) Structural comparison of linker-NE region (**C**), index (**D**), region B-C (**E**), and PE (**F**) of JMTV and *Flaviviridae* RdRPs. DENV2 (PDB entry: 6KR2) and TBEV (PDB entry: 7D6N) represent *Flavivirus*, CSFV (PDB entry: 5Y6R) represents *Pestivirus*, and HCV (PDB entry: 3HHK) represents *Hepacivirus*. JMTV structure is colored green and *Flaviviridae* structures are colored brown, except that linker and PE or its equivalent regions are shown in red and purple, respectively, and motifs B and C are shown in light cyan and grey, respectively. The α-carbon atoms of structural equivalent hallmark residues are shown in spheres. The 3500 K composite SA-omit 2*F*_o_– *F*_c_ electron density maps of JMTV RdRP was overlaid (contoured at 1.0 σ). For better clarity, the PE_C_ of CSFV and HCV RdRPs were not shown in the superimposition (left), but were shown in the individual structure. Superpositioning is fingers-based for panels C-D, palm-based for panel E, and RdRP-based for panel F.

To analyze distinct structural characteristics within JMTV NSP1 RdRP, we conducted maximum-likelihood superpositioning (see Materials and Methods) of the JMTV structure and three selected flavivirus RdRP structures. This analysis encompassed the entire RdRP as well as individual domains, as depicted in Figure 2. As anticipated, the palm domain demonstrates the greatest degree of structural preservation when comparing both global and domain-based superpositioning methods. Additionally, domain-based superpositioning reveals a higher level of structural uniformity, as evidenced by the lower root-mean-square deviation (RMSD) values depicted in Figure 2A. Notably, this consistency is particularly pronounced in the fingers and thumb domains, as illustrated in Figure 2, panels A and B. In accordance with previously documented viral RdRP structures, these findings indicate that the presence of rigid-body or hinging movement at the domain level is a characteristic commonly observed in this particular category of right-hand polymerases.

These superimpositions also served to emphasize several noteworthy substructures, namely the linker-NE region, index finger, the inter-domain region between motifs B and C (referred to as region B-C, also indicated by an asterisk in Figure 1C), and PE. To enhance our comprehension of the structural characteristics from an evolutionary standpoint, we broaden the scope of our analysis by incorporating the structural overlays of CSFV and HCV, which serve as representatives of the *Pestivirus* and *Hepecivirus* genera within the *Flaviviridae* family (Figure 2C–F). The JMTV NE exhibits structural consistency with flavi- and pesti-viruses (7,11,43,44). However, it is worth noting that the linker region in JMTV NE is approximately 10 residues longer compared to that of flaviviruses, and it forms a cofold with the palm domain. The linker within flavivirus NS5 is of utmost importance in facilitating comprehensive conformational alterations, thereby enabling distinct positioning of MTase in relation to RdRP. Consequently, the elongated linker present in jingmenvirus NSP1 may permit more extensive global conformational alaterations. The encirclement of the right-hand structure of the viral RdRP is primarily facilitated by hydrophobic interactions occurring between the index tip and the thumb. The results revealed that the index tip of JMTV RdRP exhibited a larger size compared to the index tips of RdRPs belonging to the *Flaviviridae* family (Figure 2D). Region B-C was proposed in our previous studies as a variable region in both length and structure that could mediate protein-protein interactions (21,45). Among the representative structures of *Flaviviridae* RdRP, it is observed that the flavivirus region B-C exhibits the greatest length, with DENV2 having 44 residues. Following this, CSFV (14) possesses 26 residues, while HCV has 18 residues (14). The length of the region B-C in JMTV, which consists of 35 residues, is situated between the corresponding lengths observed in flaviviruses and CSFV, as depicted in Figure 2E. The dual functionality of the PE has been observed in DENV2, where it is involved in both the initiation and elongation stages of RdRP activity through a process of refolding (24). In flaviviruses, the PE region is characterized by its placement between two thumb helices, constituting a single segment. However, in pestiviruses and HCV (19,29), the PE region is comprised of two distinct segments: the N-terminal segment (PE_N_) and the C-terminal tail (PE_C_) (19,29). The JMTV PE exhibits partial disorder, but it is characterized by a one-segment structure that closely resembles the PEs of flaviviruses in terms of length and structure (see Figures 1E and 2F). Although there is no apparent sequence similarity observed throughout the entire PE, two important aromatic residues (represented by dashed lines in Figure 1E), which are crucial for RdRP initiation and elongation in DENV2, appear to be conserved among flavi- and jingmen-viruses. This suggests that these two groups of viruses may share a similar mechanism of PE refolding. When considering JMTV RdRP, it can be observed that its overall characteristics bear the closest resemblance to the RdRPs found in flaviviruses. However, it also exhibits certain similarities to RdRPs present in other members of the *Flaviviridae* family. The observed similarities in the NE and region B-C between JMTV and pestvirus CSFV RdRPs indicate a potential evolutionary relationship of jingmenvirus beyond the *Flavivirus* genus, specifically in terms of their RdRPs.

### JMTV NSP1 synthesizes characteristic 3-mer abortive products and its MTase contributes to complex stability during transition from initiation to elongation

**Figure 3.**
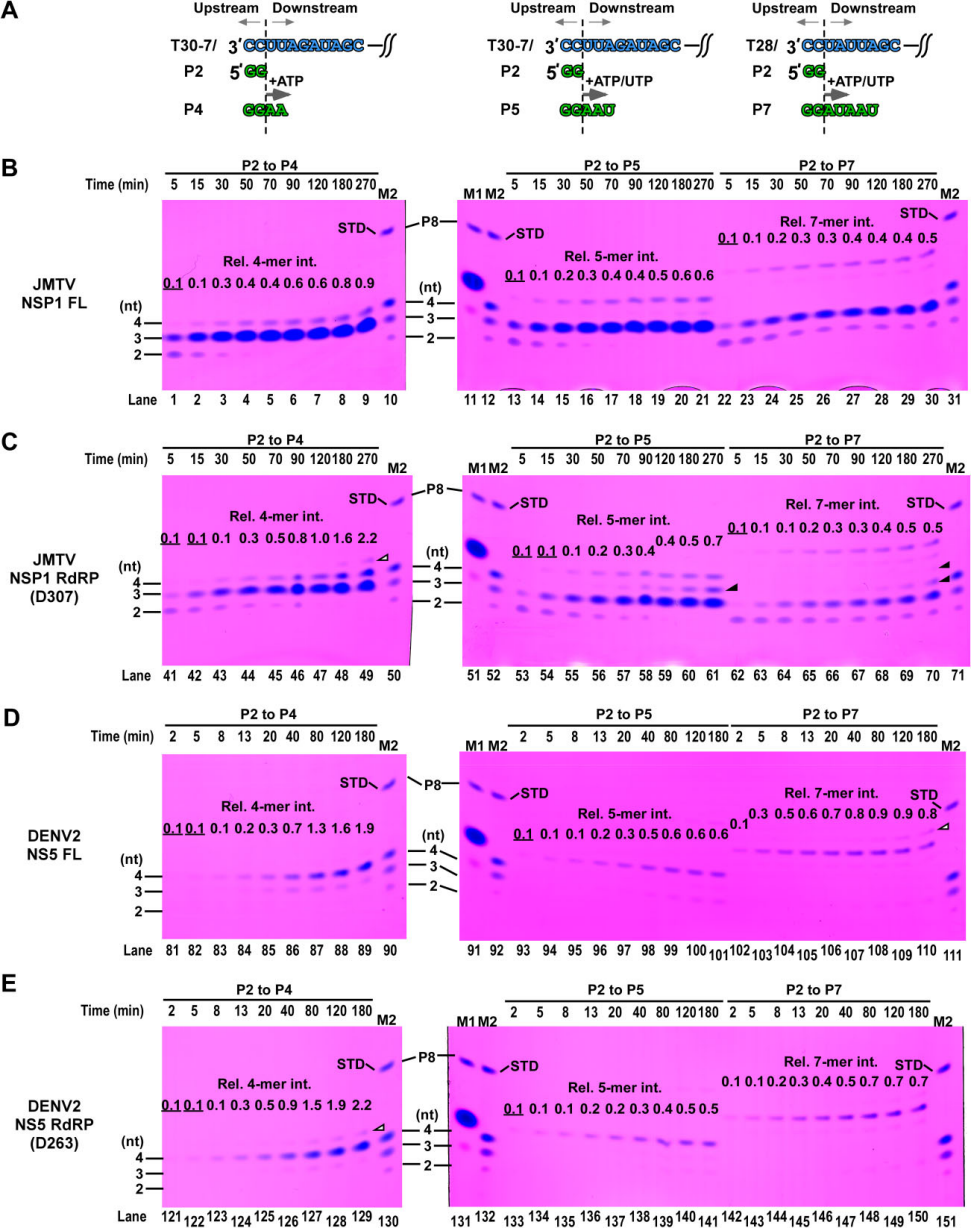
Probing the transition from initiation to elongation for JMTV and DENV2 RdRPs. (**A**) Two RNA constructs used for synthesis of RNA target lengths of 4, 5 and 7 nucleotides. (**B–E**) Denaturing PAGE analysis of time-course reactions for three different target product RNA lengths (P4, P5 and P7) for JMTV NSP1 FL/D307 mutant (B, C) and DENV2 NS5 FL/D263 (D, E). Chemically synthesized P2/P3/P4 (5′-phosphate) and P8 (5′-hydroxyl) were used as migration markers (M1: P8; M2: P2/P3/P4/P8). The molar ratio of P8:P4:P3:P2 in M2 is 1:2.5:2.5:10 and the P8 in M2 is used as the primary quantitation standard (STD, relative intensity is set to 1) for relative 4-/5-/7-mer intensity quantitation. The 0.9 relative intensity of P4 corresponds to about 1.8-fold of the template RNA molar amount, therefore suggesting multiple-turnover RdRP synthesis. The mismatch-derived products (marked by empty triangles) were included in target product quantitation. Underlined values indicate actual relative intensity values smaller than 0.1. All experiments were done in triplicates (Supplementary Figure S3) and gels from one experimental set are shown.

To gain insights into the enzymatic characteristics of JMTV RdRP and the regulatory effects of MTase on RdRP, we employed RdRP assays that were previously developed for DENV2. Specifically, we conducted a comparative analysis between JMTV NSP1 and DENV2 NS5, along with their respective MTase-deletion mutants (NSP1 D307 and NS5 D263) (24) (see Figure 3 and Supplementary Figure S3). The RNA construct T30-7/P2 consists of a 30-nucleotide template (T30) and a dinucleotide primer with a 5′-phosphate (P2). This construct is responsible for guiding the synthesis of either a 4-nucleotide product (P4) or a 5-nucleotide product (P5) when ATP or ATP/UTP are used as the sole nucleoside triphosphate (NTP) substrates, respectively (refer to Figure 3A, left). In a similar manner, the T28/P2 construct facilitates the formation of a 7-mer product (P7) exclusively when ATP and UTP are provided as the sole NTP substrates (Figure 3A, right). In order to determine whether RdRP synthesis occurs in a single- or multiple-turnover mode, a time course setting was employed. This allowed for the identification of two distinct outcomes: the formation of an unstable polymerase complex, indicative of multiple-turnover mode, or the formation of a relatively stable polymerase complex at the target product length, indicative of single-turnover mode. Regardless of the desired length of the target product, it was observed that 3-mer abortive products were more prevalent for JMTV NSP1 compared to DENV2 NS5 (as seen in panels B-C and D-E, respectively). This indicates that the complex formed by JMTV NSP1 is notably unstable when the product consists of only three nucleotides.

In the case of the P4 target product, it was observed that multiple-turnover behavior occurred for all four constructs (Figure 3, lanes 1–9, 41–49, 81–89 and 121–129; Supplementary Figure S3, back data points). As evidenced by our previous research, the utilization of Stains-All for quantifying RNA species with comparable lengths has proven to be effective (36,37). Furthermore, it has been observed that the staining efficiency of Stains-All exhibits an upward trend as the length of the RNA increases. In this study, a chemically synthesized 8-mer (referred to as P8) with hydroxyl groups present at both the 5′- and 3′-ends is employed as a standard. P8 is provided in equimolar quantities as the RNA template, and is utilized to determine the relative intensity of the band of interest. Hence, it can be observed that the relative intensity values ranging from 0.9 to 2.2, corresponding to a 4-mer band, are consistent with the occurrence of multiple-turnover synthesis at the longest reaction time. This observation is depicted in Figure 3, specifically in lanes 9, 49, 89 and 129, as further elucidated in the figure legends. In the case of the P7 target product, it was observed that all four constructs exhibited single-turnover synthesis. However, there was no apparent increase in the amount of P7 for the last three time points (Figure 3, lanes 28–30, 68–70, 108–110 and 148–150; Supplementary Figure S3, blue data points). This observation suggests that the RdRP complex has attained a higher level of stability at this particular product length. Remarkably, the quantity of intermediate products (specifically, 4-mer in the P2-to-P5 assay and 4-mer/5-mer in the P2-to-P7 assay) generated by NSP1 D307 was observed to be greater than that of FL NSP1 (as depicted in Figure 3, when comparing lanes 13–21/22–30 and lanes 53–61/62–70). This finding implies that the MTase enzyme might play a role in enhancing the stability of the RdRP during the transition from the initiation phase to the elongation phase. However, no such effect was observed in the DENV2 NS5 constructs. With respect to the relative 4-mer intensity, the two DENV2 constructs have similar levels (Figure 3, lanes 81–89 and 121–129; Supplementary Figure S3, right two panels), while the FL JMTV NSP1 has much lower level than that of the MTase-less RdRP construct (Figure 3, lanes 1–9 and 41–49; Supplementary Figure S3, left two panels), also suggesting that the MTase in JMTV NSP1 contributes to RdRP–RNA complex stability during initiation.

### JMTV NSP1 exhibits high stringency in terminal initiation and primer length

**Figure 4.**
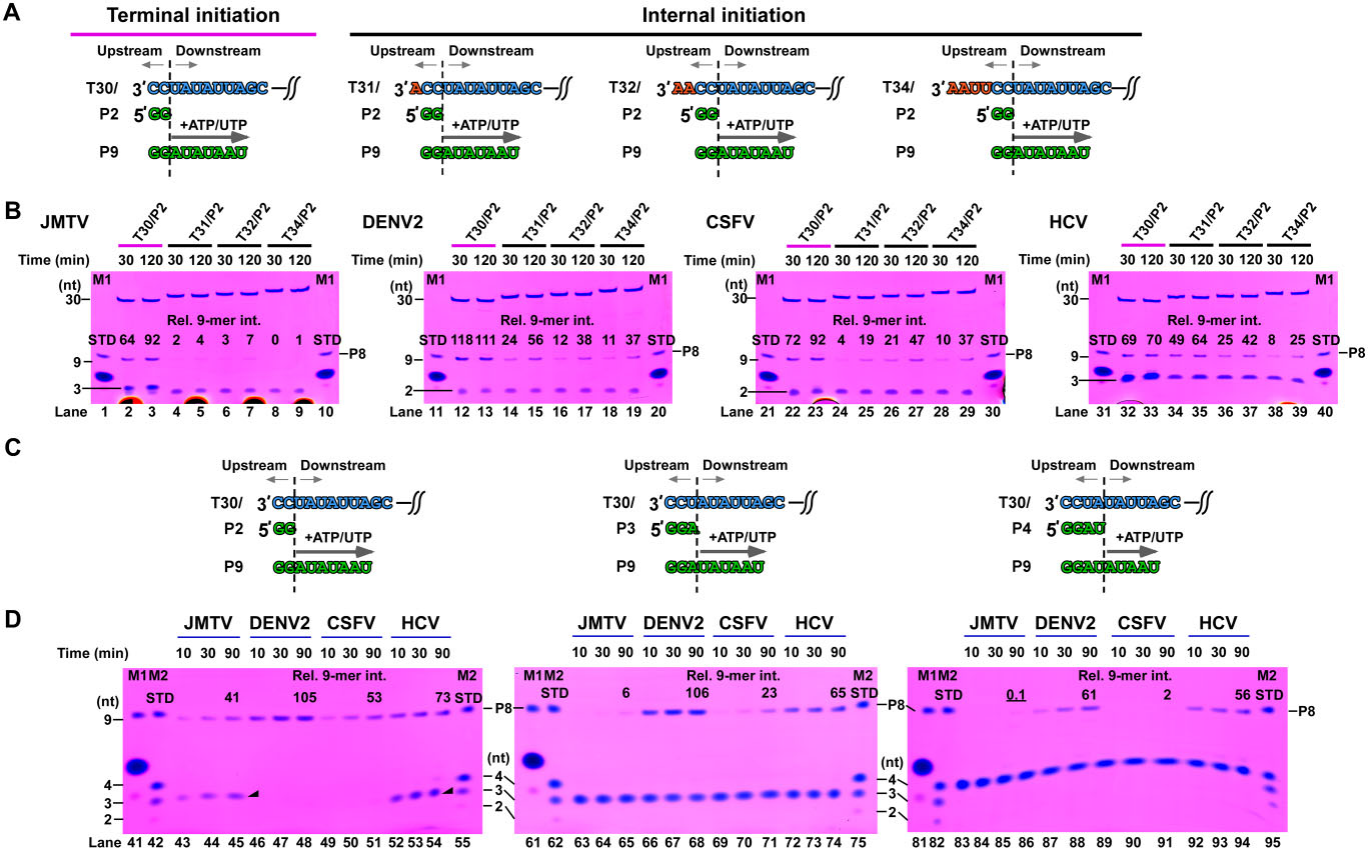
Preference assessment of terminal initiation and primer length for JMTV and representative *Flaviviridae* RdRPs. (**A**) Four RNA constructs used for terminal initiation preference assessment. (**B**) Denaturing PAGE analysis of P2-to-P9 synthesis on four different RNA constructs shown in panel A. The relative intensity of P9 is used to evaluate polymerase activities. The P8 intensity value in M1 sample was set to 100 as quantitation STD. (**C**) Three RNA constructs used for primer length preference assessment. (**D**) Denaturing PAGE analysis of PX-to-P9 (X = 2, 3 or 4) synthesis on three different constructs shown in panel C. The P8 intensity value in M2 sample was set to 100 as quantitation STD. 3-mer abortive products are indicated by solid triangles. All experiments were done in duplicates (Supplementary Figure S4) and gels from one experimental set are shown.

To enhance our comprehension of the attributes of JMTV RdRP, we conducted a comparative analysis of the terminal initiation preference exhibited by RdRPs derived from JMTV, DENV2, CSFV and HCV (Figure 4). The RNA constructs T30/P2, T31/P2, T32/P2 and T34/P2 exhibit alignment between the 5′-end of the P2 primer and the first, second, third, and fifth nucleotide of the 3′-region of the template, respectively (see Figure 4A). ATP and UTP were supplied in order to facilitate the synthesis of a 9-mer product (P9) for all four constructs. The results obtained from the analysis of four viral RdRPs indicate that the terminal initiating construct T30/P2 exhibited the highest efficiency (Figure 4B, comparing lanes 2–3/12–13/22–23/32–33 with lanes 4–9/14–19/24–29/34–39). This finding suggests that all four RdRPs have a preference for initiating replication from the 3′-end of the template. It is worth mentioning that among the four viral RdRPs that were tested, JMTV NSP1 demonstrates the greatest preference for terminal initiation. This is evident from the observation that only a minimal amount of P9 product was detected for all three internal initiation constructs (Figure 4B, lanes 4–9). The data presented indicate that the PE of JMTV NSP1 is the most sensitive to the 3′-overhang of the template RNA.

Next, we proceed to compare terminal initiation from the T30 template by utilizing primers of varying lengths, ranging from 2 to 4 nucleotides (Figure 4C, D). The results indicate that the quantity of P9 decreases as the length of the primer increases for all four RdRPs tested. This suggests that the polymerase efficiencies of these RdRPs are most suitable for *de novo* initiation or initiation driven by dinucleotides. This observation is consistent with previous structural studies on bacteriophage phi6 and HCV RdRPs (25,26). (See Figure 4D, comparing lanes 43–54 with lanes 63–74/83–94). The P2-to-P9 assay revealed that JMTV NSP1 and HCV NS5B exhibit prominent 3-mer abortive products, whereas DENV2 NS5 and CSFV NS5B do not. This observation suggests variations in the initial phases of RdRP synthesis (Figure 4D, compare lanes 43–45/52–54 with lanes 46–51). Out of all the RdRPs examined, JMTV NSP1 exhibits the greatest inclination towards shorter primers. At the maximum tested reaction time point of 90 min, the quantity of P9 derived from the P3 primer is only 15% of that derived from the P2 primer (Figure 4, comparing lane 45 with lane 65). In contrast, the other three RdRPs exhibited at least 43% of P9 quantity (Figure 4, comparing lanes 48/51/54 with lanes 68/71/74). In conjunction with the observation of the highest preference for terminal initiation, the aforementioned data indicate that the NSP1 PE of JMTV may possess the least degree of flexibility during the initiation process.

## Discussion

**Figure 5.**
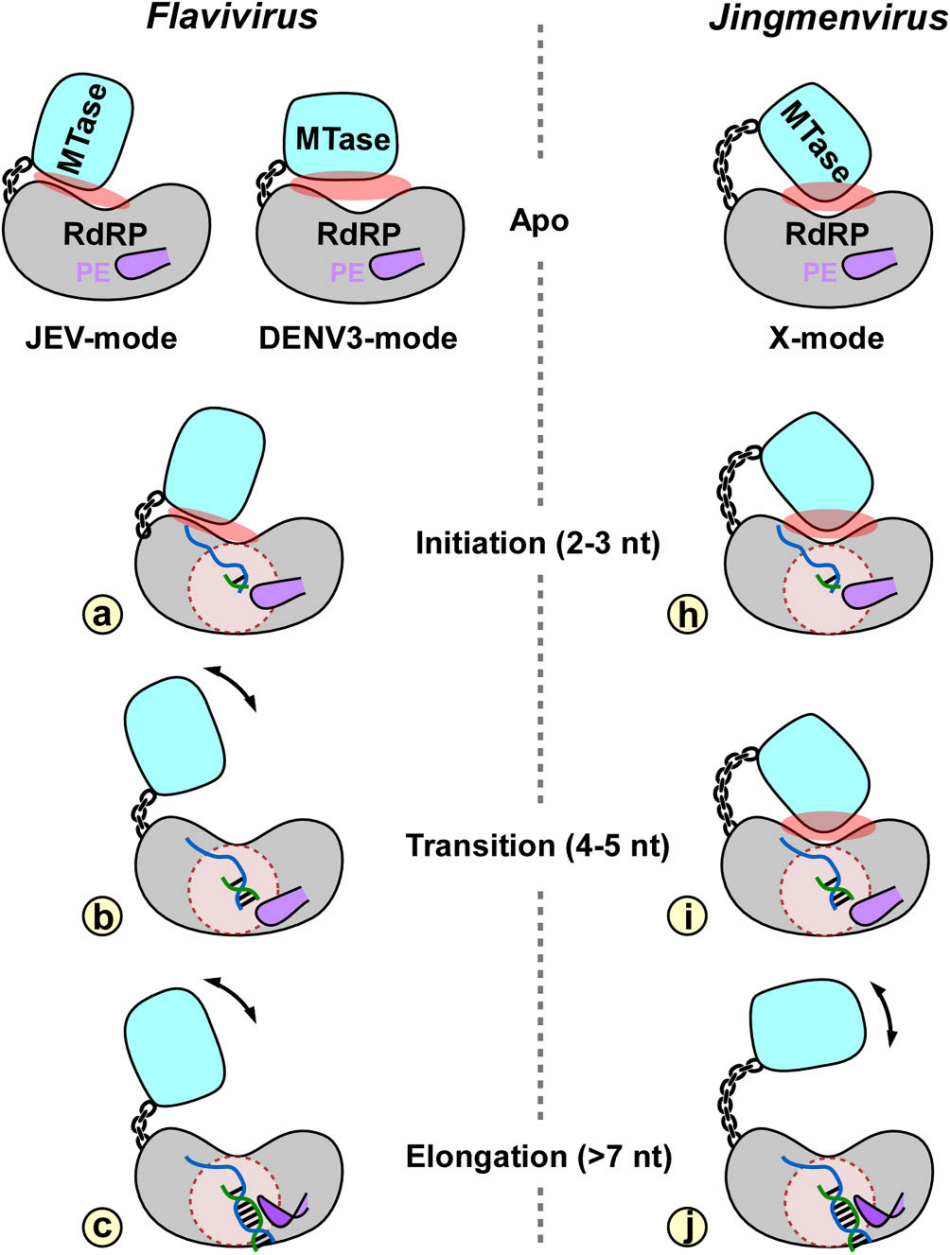
Working models for the transition process from initiation to elongation in flavivirus and jingmenvirus RdRPs. Top row: apo flavivirus NS5 can adopt two different global conformation modes (JEV-mode and DENV3-mode); on the other hand, apo jingmenvirus NSP1 global conformation is unknown (X-mode) and is likely different from those of NS5. At initiation, both enzymes take advantages of MTase-RdRP interactions (JEV-mode in NS5: model a; unknown X-mode in NSP1: model h) to initiation RdRP synthesis. Only NSP1 needs to maintain the MTase-RdRP interactions during the transition process (highlighted by model i). Upon elongation, both enzymes no longer require the initiation mode MTase-RdRP interactions. The subtle movement and shape change of the PE symbol indicate that incremental and abrupt conformational changes likely occurring in early and late stages of the transition process, respectively.

In this study, we characterize a jingmenvirus NSP1 both in structure and in RdRP enzymology. In agreement with the highest level of sequence similarity, NSP1 structurally most resembles flavivirus NS5. Although the MTase structure of NSP1 has not been solved, the RdRP structure is largely consistent with flavivirus NS5 with respect to global conformation as well as the majority of substructures. The folding of NE in jingmenvirus is shared with flaviviruses and pestiviruses, while the highest similarity of the variable region B–C is also observed between jingmenviruses and pestiviruses/flaviviruses. The enzymology data highlights the 3-mer abortive products, the strict preference of terminal initiation and shorter primer utilization by JMTV NSP1, and the role of MTase in stabilizing the transitioning RdRP complex. While the synthesis of 3-mer abortive products is shared by HCV NS5B and JMTV NSP1, the *Flaviviridae* equivalents do not match the strictness of NSP1 in terminal initiation and primer length preference. Even though jingmenvirus NSP1 and flavivirus NS5 share the MTase-RdRP layout, the MTase regulation on RdRP in these two virus groups seems to be different in several ways. Two global conformation modes have been observed in apo NS5, termed JEV-mode and DENV3-mode according to the virus system of its first identification (11,27,37). These conformation modes are depicted in Figure 5, specially in the top left models. Nevertheless, the global conformation of jingmenvirus NSP1 remains unknown due to the absence of a FL structure. The hallmark MTase-RdRP interactions in both conformation modes, more specifically the hydrophobic interactions involving RdRP index, middle, and ring fingers (key residues indicated by solid triangles in Figure 1E) in the JEV-mode and polar interactions involving index finger/nuclear localization sequence (NLS) helix in the DENV3-mode, do not include obvious sequence similarity with the corresponding regions in jingmenvirus NSP1. Hence, the details of MTase-RdRP interactions in jingmenviruses may be different (Figure 5 top, ‘X-mode’). This hypothesis may be further supported by the fact that the MTase-RdRP linker in JMTV NSP1 is much longer than the 10-residue linker in flavivirus NS5. Among the two apo conformation modes, the JEV-mode likely represents the conformation optimal for flavivirus RdRP initiation (Figure 5, model a), since mutations of key hydrophobic interface residues increase the apparent *K*_M_ for initiating NTP by three fold and the interactions help stabilize the folding of NTP-triphosphate binding ring finger (11,37). Compared with the FL NSP1, the MTase-less D307 construct produces more intermediate products of 4–5 nucleotides in length, suggesting MTase-RdRP interactions may be retained and therefore contribute to complex stability during the transition from initiation to elongation (Figure 5, models h and i). Once an elongation complex is formed, the original MTase-RdRP interactions (as in models a and h in Figure 5) are likely not required as demonstrated in DENV2 NS5 at least in the context of MTase-RdRPinterplay. A recently reported structure of a DENV2 NS5–RNA–NS3 complex shows a three-way interaction mode with both MTase and RdRP interacting with NS3 helicase domain 3, while MTase and RdRP do not form an intra-molecular interface except for being connected by the linker (15). Indeed, the release of original initiation-stage MTase–RdRP interactions may be a requirement to form such a multi-component architecture for an elongation-stage RTC.

Flaviviruses contain a large number of arthropod-borne human pathogens covering all biosafety levels (BSLs), including BSL-2 dengue viruses and Zika virus (ZIKV), BSL-3 tick-borne encephalitis virus (TBEV) and West Nile virus (WNV), BSL-4 Omsk hemorrhagic fever virus (OHFV), etc. The global-wide distribution of flaviviruses further emphasizes their threats to human beings. Although not yet indexed by the International Committee in Virus Taxonomy (ICTV), jingmenviruses have also drawn much attention due to their wide geographic distribution, closest evolutionary relationship to flaviviruses, and capability and potentials of being transfected by invertebrate vectors similar to flaviviruses. In recent years, nucleoside/nucleotide analog (NA) drugs targeting viral RdRPs have been developed for treatment of hepatitis C (46), influenza (47), and more recently, coronavirus disease 2019 (COVID-19) (48,49). However, no effective antivirals against flaviviruses or flavi-like viruses have been approved. Several NA compounds, including adenosine analogs NITD008 and galidesivir, guanosine analog AT-752, nevertheless showed promising effect against one of more DENV serotypes. With modifications at ribose 2′-position, the NTP forms of NITD008 and AT-752 both showed chain-terminating feature (50,51), while the iminoribitol *C*-nucleoside galidesivir showed hindrance of RdRP synthesis typically after consecutive incorporation (52). However, mechanism of action for these compounds has not been fully elucidated, thus hindering rational design and optimization building on these compounds. The jingmenvirus RdRPs, as illustrated for JMTV NSP1 in this study, are excellent systems in comparative study for the evolutionarily most related *Flaviviridae* RdRPs to help develop NA inhibitors either specific for jingmenviruses or broad-spectrum enough for members of the *Flaviviridae* family. The distinct enzymatic features of NSP1 may also help the study of NA drug intervention mechanisms as well as the development of jingmenvirus specific inhibitors.

In summary, through structural and enzymology characterization of JMTV RdRP, our work paves a way for further understanding the replication/transcription process of the emerging population of jingmenviruses, as well as viral RdRPs utilizing *de novo* synthesis mechanism.

## Supplementary Material

gkae042_Supplemental_File

## Data Availability

Atomic coordinates and structure factors for the reported crystal structures of JMTV NSP1 D55 and D307 have been deposited in the Protein Data bank under accession numbers 8WIL and 8WIM,respectively.
