# Supplementary material for: A jingmenvirus RNA-dependent RNA polymerase structurally resembles the flavivirus counterpart but with different features at the initiation phase
Source: Nucleic Acids Res. 2024 Jan 31;52(6):3278–90. doi: 10.1093/nar/gkae042 (PMC11014250; doi:10.1093/nar/gkae042)
Supplement: gkae042_Supplemental_File [file gkae042_supplemental_file.pdf]

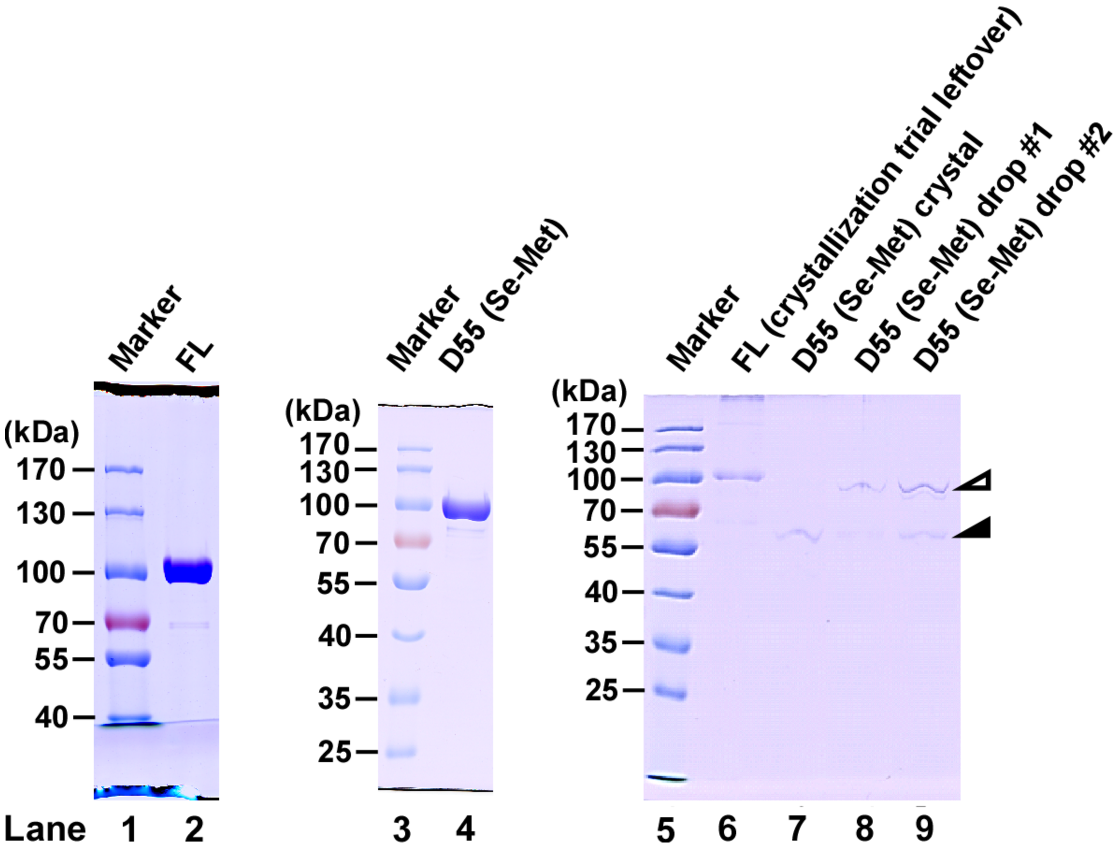

Figure S1. Crystallized NSP1 D55 is a degraded protein. Left: Purified full-length (FL) JMTV NSP1 (lane 2) migrated slightly slower than the 100 kDa protein in the marker sample (lane 1). Middle: Purified JMTV NSP1 D55 (Se-Met-substituted, lane 4) migrated about the same as the 100 kDa protein in the marker sample (lane 3). Right: JMTV NSP1 D55 (Se-Met-substituted) crystal (lane 70) migrated between the 70 kDa and 55 kDa proteins in the marker sample (lane 5); Two crystal-less drops collected after a D55 (Se-Met) crystallization screening trial indicate degradation of the D55 protein during the screening (lanes 8-9), and a primary degraded band (indicated by solid triangle) migrated about the same as the D55 crystal sample. Lane 6: Leftover FL NSP1 sample after a crystallization screening trial. Note that the D55 protein migration difference observed in lanes 4 and 8/9 may be related to the difference in solution conditions.

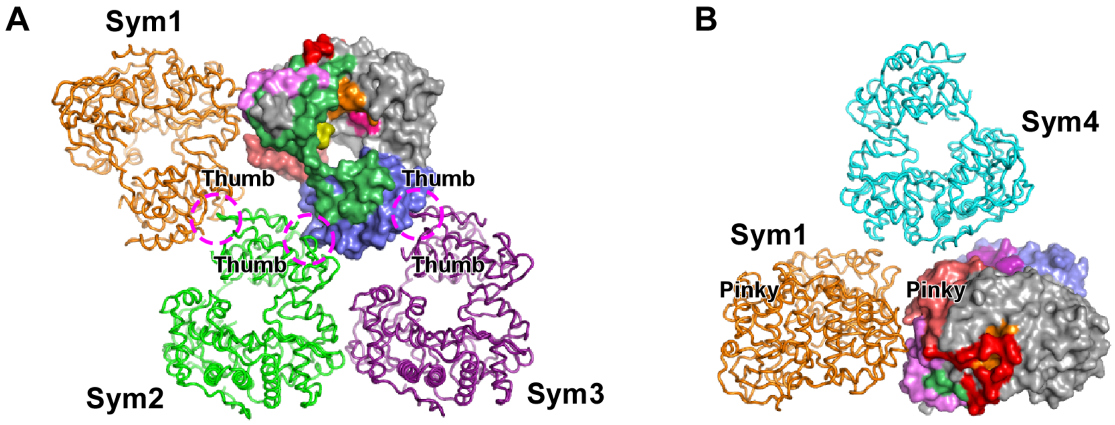

Figure S2. Crystal packing interactions involving thumb and pinky finger in the JMTV NSP1 D307 structure. A) The thumb domain of JMTV NSP1 D307 is involved in periodical interactions between the top of the thumb and the bottom of the thumb from a symmetry related molecule (indicated by dashed pink circles). The tip of thumb (α26-α27 loop in Figure 1C) is buried in a pocket at the bottom of the neighboring thumb. B) By contrast, no extensive interactions are observed between the pinky finger and its neighboring molecules (Sym1 and Sym4). The D307 structure is shown in surface representations and color-coded as in Figure 1C. The symmetry related molecules (Sym1/Sym2/Sym3/Sym4) are shown as loop representations and in different color. Same zoom level is used for both panels.

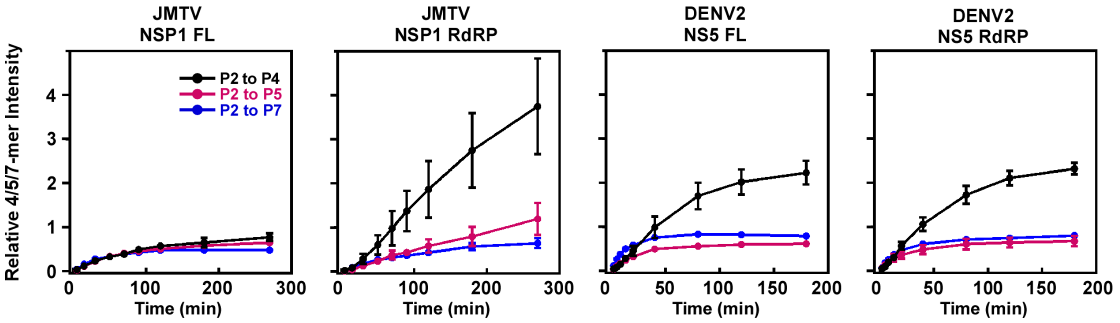

Figure S3. Quantitative analyses of target products in RdRP assays for assessment of the transition from initiation to elongation. Relative 4-mer (black), 5-mer (red), and 7-mer (blue) intensity values from triplicated experiments were quantified and average number and standard deviation values are shown as points and error bars, respectively. Gels from one experimental set are shown in Figure 3.

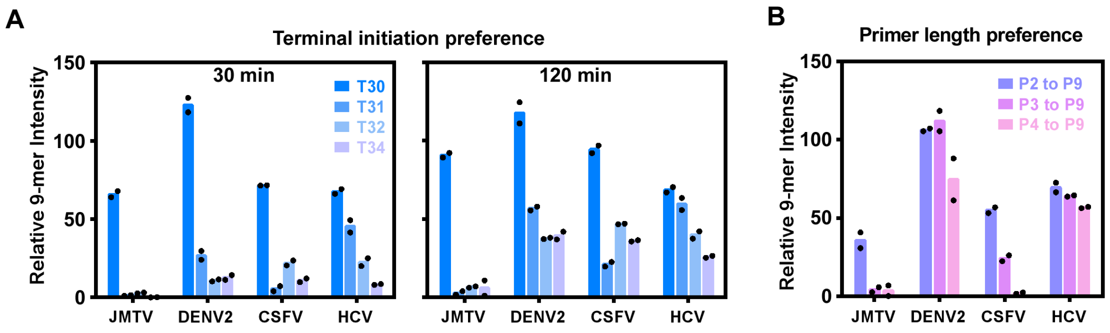

Figure S4. Quantitative analyses of terminal initiation and primer length preferences. A-B) Relative 9-mer intensity values were quantified from duplicated experiments, and average number and actual relative intensity values are shown as vertical bars and dots, respectively. Gels from one experimental set are shown in Figure 4, B (corresponding to panel A of this figure) and D (corresponding to panel B of this figure).
